# The excessive celebrity worship behavior questionnaire: Chinese scale development and validation

**DOI:** 10.1371/journal.pone.0303683

**Published:** 2024-05-22

**Authors:** Yiqing He, Qinxue Liu

**Affiliations:** 1 School of Education, Tianjin University, Tianjin, China; 2 School of Psychology, Central China Normal University, Wuhan, China; Shandong Jiaotong University, CHINA

## Abstract

**Objective:**

To develop a scale to assess the excessive behavior of superfans in celebrity worship and to test its reliability and validity in China.

**Methods:**

The scale was developed based on the netnography and interviews of celebrity fans and the existing problematic Internet usage scales. Sample 1 (n = 465) was used for exploratory factor analysis, and Sample 2 (n = 804) was used for confirmatory factor analysis, reliability test, criterion validity, and discriminative validity test.

**Results:**

There were 36 items in the final scale, including nine factors: impaired social functioning, replacement of real to virtual social relationships, sleep and eating problems, withdrawal, mood alteration, salience, excessive buying, increased craving, and escape from real life. The factor loadings ranged from 0.565 to 0.803. Confirmatory factor analysis showed that the scale was well structured. The reliability of the scale and each factor were satisfactory. The scale showed good discriminant validity in reflecting celebrity worship behavior. In terms of scores, mood alteration was the highest, excessive buying was the lowest, and there were certain gender and age differences.

**Conclusion:**

This study initially identified the main characteristics of excessive celebrity worship behavior among young fans on social media. The developed Chinese scale has good reliability and validity and can be used as a measurement tool.

## 1. Introduction

In recent years, given the highly developed digital platforms and social media, fans have become involved in celebrity worship behavior and fandom culture in a broader and deeper sense in East Asia [1, 2]. Nevertheless, online fandom culture in China has been frequently criticized recently due to the concern that it may give rise to various negative social issues. For example, fans may initiate extreme cyber violence on those who criticize their idols, massive online fighting is frequently provoked and organized between fan groups, leader fans constantly make requests of compulsive labor and buying on follower fans, and some young fans commit self-injury behavior or even try to commit suicide [3–5].

As reported in previous research, excessive celebrity worship is associated with several types of behavioral addictions that reflect obsession, compulsion, or intrusion [6, 7], and significantly lead to negative consequences in youth, such as diminished self-efficacy, self-esteem, intellectual interest, work or study performance, and identity achievement [8–11], commodification and objectification of the human being [12], depression, anxiety, social dysfunction, neuroticism, somatic symptoms, negative affect, dissociation,poorer mental health and psychological well-being [6, 13], addictive tendencies [14], problematic Internet and social media use [15, 16], delinquency and substance abuse [7, 17], stalking behavior and compulsive buying [18, 19]. In view of this, the current study aims to develop a new scale to measure excessive celebrity worship driven by fandom culture in the new media era.

### 1.1 Literature review

In previous studies, celebrity worship was defined as extremely profound adoration and devotion to a celebrity [20], or an unreciprocated, parasocial relationship characterized by fantasies, preoccupation, psychological identification, and emotional attachment [21, 22], or establishing a solid identity and gaining a sense of fulfillment in relationships featuring psychological absorption and addiction [14], or intense, compulsive and obsessional attachment/involvement characterized by primary focus, strong identification, devotion, loyalty and investment [23–25]. These studies linked the absorption of young fans with addiction to celebrity worship [22, 26], in that celebrity worshippers show tolerance, craving, obsession, compulsion, preoccupation, etc. The evolution of absorption along the rank of celebrity worship may stimulate psychological needs in the form of addiction. Tolerance to the milder levels of celebrity worship motivates an individual to reach the deeper, more pathological levels to satisfy the addiction, bringing about ‘Borderline-pathological’ attitudes [14, 25, 27, 28]. The extreme kind of celebrity worship encompasses obsessive compulsive and even delusional behaviors online, which were closely correlated with problematic Internet use.

Despite the absence of a consensual definition, problematic Internet use is mainly defined as a maladaptive pattern of Internet use, generally time-consuming, which results in critical impairment or distress clinically [29, 30]. It is mainly characterized by the inability or difficulty to control the time spent online [31, 32], which is linked to negative behavioral, psychosocial or physical consequences [33]. Therefore, this study attempts to define excessive celebrity worship behavior from the perspective of addiction: fans are excessively obsessed with the celebrity by investing too much time and resources online, with loss of self-control, increased impulsivity, and withdrawal symptoms when interrupted. This excessive state exerts an adverse impact on psychological well-being, study, work, and social functioning [7, 13, 17, 19, 26].

Existing research has adopted diverse scales to measure celebrity/idol worship, including the Celebrity Worship Scale [14], Celebrity Attitude Scale (CAS, the revised form of CWS scale) [13, 14, 28], the Idol Worship Questionnaire [21], Expression of Idolization Scale [20], Obsessive Relational Intrusion and Celebrity Stalking Scale [18], Celebrity Appeal Questionnaire (CAQ) [23], a short form of the Relationship Rating Form [34], Parasocial Interaction Scale (PSI) [35], Sport Fan Motivation Scale (SFMS) [36], the Public Figure Preoccupation Inventory [7] and unique scales developed for the specific studies.

Currently, the most widely used and analyzed scale is the Celebrity Attitude Scale (CAS) [14] and its revised versions [25]. The scale was developed based on the absorption-addiction model and covered three perspectives: entertainment–social, intense–personal, and borderline–pathological. Among them, only a few items in the intense-personal and borderline-pathological components manifest excessive behavior. The Idol Worship Questionnaire measures identification-emulation idolatry (IEI), which consists of five components: attachment, consumption, identification, idealization, and romanticization [9]. IEI focuses on the normal part of identity formation during celebrity worship and considers it beneficial for psychological development. The Obsessive Relational Intrusion and Celebrity Stalking Scale mainly rates the offline behavior of sasaeng fans who focus on the private life of the idol, not online addictive behavior [37, 38]. At present, there is no scale specifically designed to systematically measure excessive and problematic behaviors for contemporary idol culture on social media.

In the meantime, with the emergence of new media technology, the development and adaptation of measurement tools for Internet use have shown rapid growth. Previous measurement scales developed for Internet Addiction and its related constructs (e.g., problematic Internet use, pathological Internet use, mobile phone addiction, problematic social media use, and mobile phone dependence) have identified several common characteristics of addictive behavior including salience, loss of control, compulsive use, tolerance, craving, escape, social comfort, mood change, and so forth [39–42]. This reveals that excessive behavior in celebrity worship may also involve multidimensional addictive presentations. Consequently, these scales lay a solid theoretical basis for the development of a new questionnaire that mainly reflects the problematic aspects of idolatry. They provide a new theoretical perspective of behavioral addiction in evaluating and describing the excessive behavior of frenetic fans.

### 1.2 The current study

Based on the literature review above, this study intends to develop a new scale to measure excessive celebrity worship for fandom fans from the theoretical perspective of behavioral addiction. First, a preliminary questionnaire was developed in pre-test, and then two samples were collected for Exploratory factor analysis and confirmatory factor analysis, respectively. Descriptive statistical analysis, item analysis, reliability test, exploratory factor analysis, criterion validity, and discriminative validity test were performed to validate the final scale.

This new scale can reach the following targets: (1) Evaluate the excessive state of worship behavior in a quantitative way, especially in young fans. This scale can serve as a measurement tool for the government, social media platforms, pop culture industry, schools, parents, and the fans themselves to assess the extent of the addictive nature of celebrity worship behavior in the fan community. (2) Identify the multi-dimensions of excessive celebrity worship behavior. By exploring the factors, it can be inferred that the underlying psychological and neural mechanisms of excessive celebrity worship behavior might be highly similar to that of problematic Internet use. (3) In future studies, explore the antecedents/outcomes of excessive celebrity worship quantitatively. For example, how deep involvement in the fan community influences students’ academic performance and life well-being can be examined with a large sample.

This scale will be employed on celebrity fans. The extent of excessive behavior will be evaluated as a continuum, which means there is currently no threshold for distinguishing “excessive fans” and “non-excessive fans”. The development and application of this new scale can open up a new stream of quantitative research in fan studies and problematic behavior studies. With the application of this questionnaire, the government, social media platforms, schools, and parents of young fans will be clearly aware of the maladaptive behavior in the fan community and what act to take next step accordingly. Young fans themselves can also assess their addictive state to decide whether withdrawal is needed. As a result, this scale has extensive practical implications.

## 2. Methods

### 2.1 Preliminary questionnaire

Netnography and interviews were conducted on the three most popular social media platforms in the pop culture community (Weibo, WeChat, and Douban). Six fan groups and 107 fans were tracked and observed in depth longitudinally, 28 of whom participated in semi-structured interviews. The interviewees were asked questions like” How long have you been feeling obsessed like this?”“Did you do anything special for this idol?”“Is there anything different after becoming your idol’s super fan?”“Does the deep love affect your life?”, etc. Consequently, a rich database of first-hand qualitative data was created. Several problematic presentations were in the original database: salience, craving, withdrawal, social comfort, and interference with daily life. *"I can’t control myself at all; sometimes I can’t help but pick up the phone to have a look when I drive and walk or even wake up in the middle of the night*.*" “I heard that she had spent hundreds of thousands of RMB Yuan on him*, *and much of it was borrowed from others*. *Now she refuses to pay back and threatens to self-destruct*.*" "My roommate ate very little these days*. *She cried in the room every day and quarreled with us whenever we brought up critiques*. *She is totally out of her mind*.*" "Recently*, *I stared at my mobile phone every day from morning to night*. *My eyes were so sore that I couldn’t even see clearly at night*.*"* According to the conceptual definition, potential items were identified from the original transcripts and were labeled by their theme. Dimensions and concepts in existing scales measuring problematic Internet/smartphone use were considered in identifying and labeling the items to match the addictive and obsessive nature of excessive celebrity worship. A library was established with the extracted potential items. These items were classified according to their themes and similar items were combined together to form a final one. Finally, a preliminary questionnaire with 80 items was developed and scored on a Likert five points ranging from 1 (very inconsistent) to 5 (very consistent). Face validity refers to the degree to which a test respondent views the content of a test as relevant for the situation being considered,as judged by the individual answering the item [43, 44]. Experts, researchers, graduate students, and fans were invited to confirm face validity during the development of the scale. In the pre-test and feedback, the participants all confirmed the relevance of the questions regarding excessive or problematic celebrity worship, agreed that the various items captured the essential information of our construct and the items were recognisable and generally easy to answer [45, 46]. The participants offered several constructive suggestions, e.g. using the term “star-chasing” which is easier for fans to understand in Chinese.

### 2.2 Participants

Ethical approval was obtained from the ethics committee of Tianjin University. Written informed consent was confirmed from all participants, including parents or guardians of the minors. The participants were recruited in June 1^st^ -December 15^th^, 2021. Sample 1 was used for item and exploratory factor analyses—the preliminary questionnaire comprised 80 items. In total, 465 valid responses were obtained from 498 respondents. There were 114 males, 351 females, 36 respondents aged 20 and below, 64 aged 21–23, 135 aged 24–26, 179 aged 27–30, and 51 over 30 years. Among the respondents receiving higher education, 75 had studied or were studying in top-tier universities in China (“First-class universities and disciplines of the world”), 338 in other domestic universities and colleges, 17 in higher vocational colleges, 6 in overseas universities, 8 in research institutes, and 21 in secondary or high school. (see S1 Dataset).

Sample 2 was used for confirmatory factor analysis, reliability testing, criterion validity, discriminative validity testing, and score analysis. A total of 804 valid questionnaires were collected from fans on social media platforms. The criterion variable was the Chinese revised version of the Idol Worship Questionnaire, the average daily time spent and frequency on social media for celebrities, including 183 males and 621 females. 72 respondents aged 20 and below, 132 aged 21–23, 230 aged 24–26, 274 aged 27–30, and 96 over 30 years. 128 people had studied or are studying in top-tier universities in China (“First-class universities and disciplines of the world”), 584 in other domestic universities and colleges, 35 in higher vocational colleges, 7 in overseas universities, 12 in research institutes, and 38 in secondary or high school. After two weeks, the formal scale was retested to calculate the test-retest reliability using a sample of 94 respondents. (see S2 Dataset).

### 2.3 Statistical methods

SPSS (version 20.0) was used for descriptive statistical analysis, item analysis, reliability test, exploratory factor analysis, criterion validity, and discriminative validity test. Confirmatory factor analysis was performed using EQS 6.1.

## 3. Results

### 3.1 Exploratory factor analysis

Sample 1 was used for the exploratory factor analysis and dimension reduction. First, Bartlett’s statistic was significant (χ2 = 5719.852, P < 0.001), and the result of the Kaiser-Meyer-Olkin (KMO) test was satisfactory (0.898). Using principal component analysis with varimax rotation, combined with the scree plot, nine factors with eigenvalues greater than one were extracted. Items were removed according to the following criteria: factor loading was less than 0.4, at least two-factor loadings were more than 0.4, and the difference between the two highest factor loadings was less than 0.2. After several rounds of deletion, the remaining items had the single highest loading, and the other loadings were all less than 0.3. Finally, 44 items were removed, the remaining 36 items were reduced to nine factors, and the cumulative variance explained was 59.349%. Table 1 presents the results of the exploratory factor analysis.

**Table 1:** Exploratory factor analysis results of the excessive celebrity worship behavior scale.

factors								
F1:impaired social functioning	F2:replacement of real to virtual social relationships	F3:sleep and eating problemss	F4:withdrawal	F5:mood alteration	F6:salience	F7:excessive buying	F8:increased craving	F9:escape from real-life
0.65	0.71	0.76	0.71	0.65	0.80	0.80	0.70	0.59
0.63	0.68	0.69	0.64	0.71	0.76	0.76	0.67	0.74
0.66	0.68	0.62	0.69	0.63	0.80	0.80	0.60	0.76
0.69	0.62	0.75	0.65	0.65				
0.66	0.63							
0.56	0.61							

**Fig 1:**
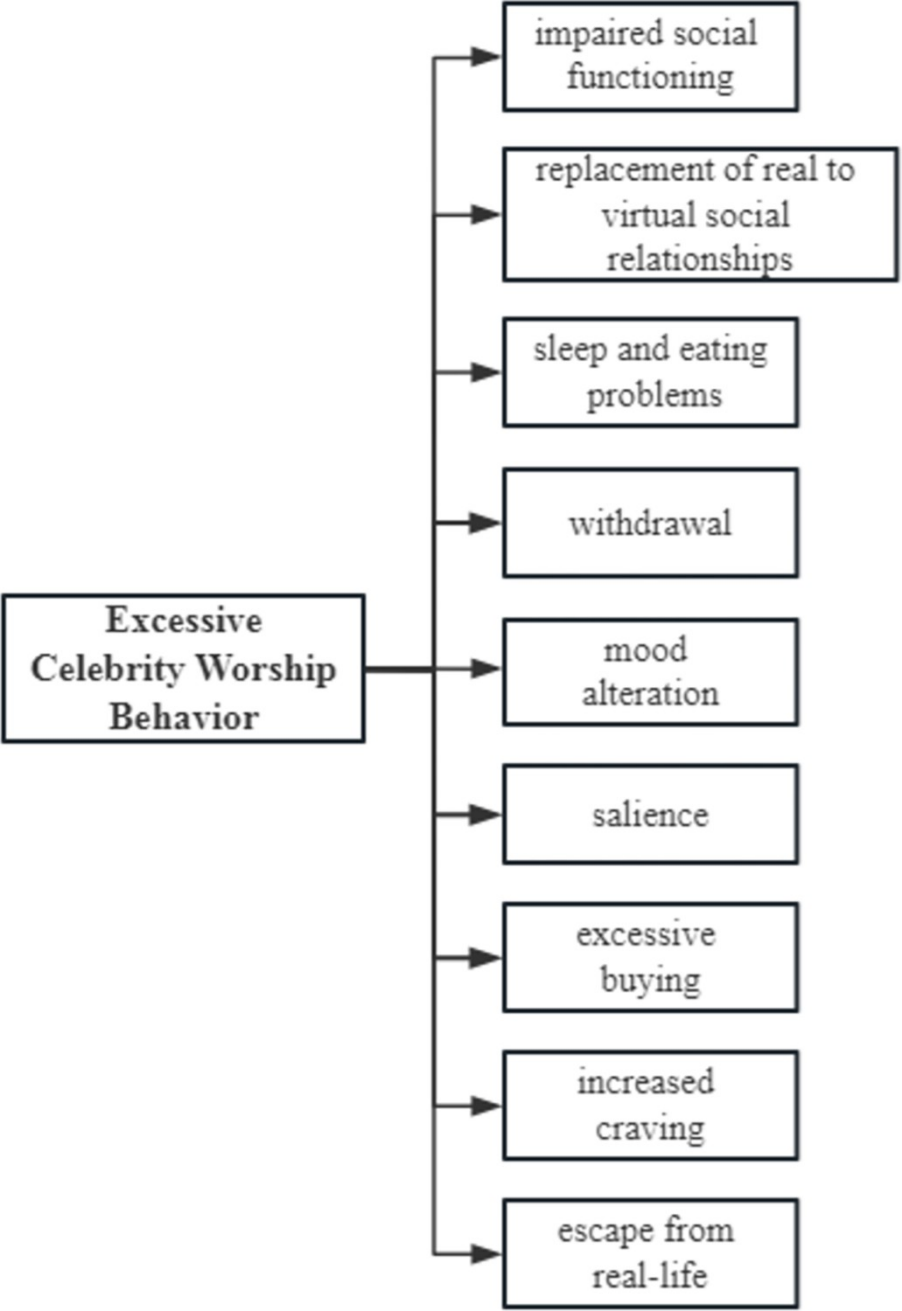
Factor framework of the scale.

Table 2 shows the factor structure and items of scale. Factor 1 is impaired social functioning, which means that excessive worship behavior interferes with daily social functions, including studies, work, and social relations, resulting in increased conflict. Factor 2 is replacement of real to virtual social relationships, which means that fans develop intimate relationships within the online fan community and feel socially recognized and relaxed in independent spaces on social media platforms, which can even replace offline social interactions. Factor 3 is sleep and eating problems, which means that being over-obsessed with celebrity worship affects basic life activities, such as sleep and eating. Factor 4 is withdrawal, which refers to negative psychological and behavioral responses (e.g., irritability, anxiety, and depressive symptoms) when fanship experiences encounter obstacles, such as the inability to access mobile phones to support idols due to work or study requirements, the spread of rumors about their idols, or loss of track of their idols on social media. Factor 5 is mood alteration, which means that fans experience positive emotional experiences and mood changes through fanship behavior. Factor 6 is salience, which means that the celebrity and fan community occupy the majority of the thinking and behaviors of a fan, showing obvious obsession and attention bias with loss of control. Factor 7 is excessive buying, which means that fans who are obsessed with their idols spend far more than a reasonable amount of economic expenditure to show support and earn recognition. Factor 8 is increased craving, which means that the desire for the fanship experience is getting stronger and stronger, so involvement in worship behavior is also growing deeper to meet the expanding psychological needs. Factor 9 is escape from real life, which refers to fans fully engaging in fanship behavior as an escapist approach to avoiding negative experiences in real life. Fig 1 illustrates the framework of this scale.

**Table 2:** Factor structure and items of the excessive celebrity worship behavior scale.

Factors	Question items
F1:impaired social functioning	I sometimes procrastinate on other things because of my obsession with my idol.
	Being a fan has caused some negative effects on my studies or work.
	I have less interaction with my family because of star-chasing online.
	After becoming a fan, I usually have less time for other leisure activities.
	I used to delay the schedule because of engaging in star-chasing.
	I often look at my mobile phone to chase my idols in inappropriate occasions, such as eating with or talking to others.
F2:replacement of real to virtual social relationships	Compared with real life companion, I prefer to make friends with online fellow fans.
	I am more respected and recognized in the online fan community than in offline real life.
	Compared with real social interaction, people in the online fan community are more accepting of me for who I really am.
	I’m more satisfied with the relationships I build online with my fan companions than with those in real life.
	Compared with my companions in real life, my online fan friends treat me better.
	Compared with socializing in real life, I feel safer communicating with my fan companions online.
F3:sleep and eating problems	On more than one occasion, I have slept very little because of chasing my idol.
	I habitually cut back on sleep so that I can spend more time following my idol.
	I feel drained of energy during the day when I stay up late online for my idol.
	I used to skip meals when I was engaging in chasing my idol.
F4:withdrawal	If I cannot access my idol online, I will feel uncomfortable and unaccustomed.
	If I cannot access my idol online, I will be restless and anxious.
	If I cannot access my idol online, I will be depressed and down in the spirit.
	If I cannot access my idol online, I feel like I’m missing something.
F5:mood alteration	Following the celebrity can alleviate my loneliness.
	I often feel that chasing idols can make my heart more peaceful.
	I feel most comfortable and relaxed when I am immersed in chasing my idol online.
	When I am following my idol online, I feel carefree.
F6:salience	Once I start chasing my idol on the Internet, I won’t think about anything else.
	When I was chasing my idol on the Internet, I almost devoted myself to it.
	I almost forget everything else when I go online to catch up with my idol.
F7:excessive buying	I have been reminded or criticized for spending too much money on supporting the celebrity.
	I spent more than I expected on star chasing.
	I once had an argument with my family over the consumption problems in fan community.
F8:increased craving	On average, I spend more time online following my idols every week than before.
	I have to spend more time online chasing my idol than before to feel satisfied.
	My inner desire for star chasing is increasing.
F9:escape from real-life	When I chase stars, I don’t have to think about the difficulties I face in life.
	I often escape unhappy things by engaging in online star chasing.
	I’m immersed in online star chasing to escape the things I don’t want to do but have to do.

### 3.2 Item analysis

The total scores for all items were summed and arranged from high to low. The highest 27% of the respondents were selected as a high-score group (n = 126), and the bottom 27% as the low-score group. An independent sample t-test was used to compare the differences between the two groups for all items. There was a significant difference between the high and low groups for each item (P < 0.001), and the range of critical ratio values was 5.7–16.0, which were all higher than 5. The standard deviation of each item ranged from 0.84 to 1.377. The item-total correlation range was 0.479–0.679 (P < 0.001). The critical ratio value and item-total correlations are presented in Table 3.

**Table 3:** Critical ratio value and item-total correlation of all items.

Item	*r*	Critical ratio	Item	*r*	Critical ratio	Item	*r*	Critical ratio	Item	*r*	Critical ratio
1	0.596***	11.781***	10	0.590***	12.527***	19	0.669***	13.952***	28	0.605***	13.672***
2	0.602***	14.362***	11	0.576***	10.994***	20	0.615***	8.742***	29	0.631***	14.839***
3	0.617***	11.862***	12	0.574***	11.318***	21	0.506***	6.187***	30	0.583***	14.940***
4	0.624***	12.340***	13	0.620***	10.520***	22	0.520***	4.370***	31	0.595***	10.048***
5	0.595***	11.348***	14	0.622***	13.804***	23	0.490***	7.890***	32	0.651***	14.960***
6	0.600***	11.972***	15	0.576***	13.405***	24	0.527***	7.281***	33	0.604***	10.873***
7	0.577***	11.662***	16	0.596***	11.699***	25	0.536***	9.830***	34	0.495***	7.222***
8	0.608***	12.465***	17	0.671***	12.756***	26	0.564***	9.963***	35	0.479***	5.735***
9	0.568***	9.743***	18	0.679***	15.952***	27	0.577***	10.750***	36	0.519***	9.311***

Note

* P < 0.05

* * P < 0.01

* * * P < 0.001, the same below.

### 3.3 Construct validity of the formal questionnaire

A total of 804 valid responses were obtained. EQS6.3 was used to conduct a confirmatory factor analysis on the 9-factor structure of the 36 items. The results are summarized in Table 4. The statistics of the 9-factor model reached a good model-fitting requirement and were significantly better than that of the 8-factor model, indicating satisfactory discriminative validity.

**Table 4:** Confirmatory factor analysis of excessive celebrity worship behavior scale.

Model	*χ2*	*df*	*χ2/df*	*RMSEA*	*NFI*	*NNFI*	*CFI*	*GFI*	*Δχ2*	*P*
Factor 9	1079.850	558	1.94	0.034	0.979	0.988	0.990	0.922		
8 Factor (F1 + F3)	1257.521	566	2.22	0.039	0.975	0.985	0.986	0.910	177.671	<0.001
8 Factor (F1 + F7)	1355.935	566	2.40	0.042	0.973	0.982	0.984	0.904	276.085	<0.001
8 Factor (F5 + F9)	1251.483	563	2.22	0.039	0.975	0.985	0.986	0.911	171.633	<0.001

As shown in Table 5, the correlation between factors was at a medium level, ranging from 0.292 to 0.698, and significant at the level of P < 0.001. The correlation between each factor and the total score was higher than the correlation within factors, indicating that the construct of “excessive celebrity worship behavior” converged as a whole and that the contribution of each dimension to the total score was strong. All statistics indicated that the construct validity of the scale was satisfactory.

**Table 5:** Correlation between each factor.

	Total score	Impaired social functioning	Replacement of real to virtual social relationships	Sleep and eating problems	Withdrawal	Mood alteration	Salience	Excessive buying	Increased craving
Impaired social functioning	0.806***								
Replacement of real to virtual social relationships	0.766***	0.488***							
Sleep and eating problems	0.746***	0.698***	0.439***						
Withdrawal	0.806***	0.574***	0.549***	0.526***					
Mood alteration	0.661***	0.345***	0.559***	0.337***	0.537***				
Salience	0.646***	0.376***	0.429***	0.360***	0.517***	0.420***			
Excessive buying	0.718***	0.641***	0.459***	0.525***	0.536***	0.292***	0.401***		
Increased craving	0.739***	0.499***	0.522***	0.441***	0.598***	0.476***	0.544***	0.493***	
Escape from real-life	0.638***	0.425***	0.408***	0.416***	0.473***	0.517***	0.404***	0.326***	0.422***

### 3.4 Reliability test

Table 6 shows Cronbach’s α, split-half reliability, and test-retest reliability. The Cronbach’s α of the total score was 0.945, and the Spearman-Brown split-half coefficient was 0.881. The reliability of each factor ranged from 0.679 to 0.853, split-half reliability ranged from 0.671 to 0.873, and test-retest reliability ranged from 0.801 to 0.922. These results show that the reliability of the questionnaire was satisfactory.

**Table 6:** Reliability analysis results.

Scale	Cronbach’s α	Split-half reliability	Test-retest reliability
Impaired social functioning	0.846	0.873	0.922
Replacement of real to virtual social relationships	0.853	0.850	0.921
Sleep and eating problems	0.821	0.803	0.883
Withdrawal	0.833	0.812	0.903
Mood alteration	0.775	0.775	0.870
Salience	0.831	0.834	0.856
Excessive buying	0.799	0.764	0.869
Increased craving	0.780	0.762	0.844
Escape from real-life	0.679	0.671	0.801
Total score	0.945	0.881	0.978

### 3.5 Criterion-related validity analysis

This scale measures the excessive behavior of fans in celebrity worship from the perspective of behavioral addiction; therefore, we adopted the revised Chinese version of the CAS scale as the criterion variable. The results showed a significant positive correlation between each dimension of the excessive celebrity worship behavior scale and the dimensions of the criterion scale, with a correlation coefficient ranging from 0.284 to 0.617 (Table 7).

**Table 7:** Criterion-related validity.

Chinese version of CAS scale		Problematic Idolatry Behavior Scale								
		Impaired social functioning	Replacement of real to virtual social relationships	Sleep and eating problems	Withdrawal	Mood alteration	Salience	Excessive buying	Increased craving	Escape from real-life
	Emotion-casted	0.467***	0.524***	0.453***	0.581***	0.538***	0.475***	0.384***	0.500***	0.515***
	Completely-identity	0.617***	0.565***	0.552***	0.607***	0.502***	0.465***	0.577***	0.549***	0.466***
	Relation fantasy	0.470***	0.529***	0.458***	0.564***	0.575***	0.444***	0.422***	0.507***	0.599***
	Borderline-pathological	0.562***	0.447***	0.498***	0.482***	0.284***	0.349***	0.527***	0.453***	0.367***
	Entertainment-social	0.422***	0.552***	0.378***	0.523***	0.635***	0.390***	0.334***	0.469***	0.399***

### 3.6 Discriminative validity

This questionnaire should be able to distinguish the different levels of excessive behavior of fans. For example, the score of super fans (i.e., "die-hard fans") should be higher than that of marginal fans (i.e., "passers-by fans"). Duration of Internet usage is one of the most predictive indicators in the assessment of Internet addiction. To examine the discriminative validity of our scale, respondents were divided into two groups (high-involved group and low-involved group) according to the score of the criterion variable, the daily average time spent on celebrity worship, and the daily average frequency of using mobile phones for celebrity worship. The independent sample t-test results for the two groups are presented in Table 8. The results showed significant differences between the two groups of fans in terms of the score for excessive behavior; the score for excessive behavior in the high-involved group was higher than that in the low-involved group, indicating that the scale had good discriminative validity. The differences were significant for both the total score and for each factor.

**Table 8:** Discriminative validity results.

	Chinese CAS			Duration of time			Frequency		
Factor	High involved group (n = 396)	Low involved group (n = 408)	*T-value*	High involved group (n = 433)	Low involved group (n = 371)	*T-value*	High involved group (n = 465)	Low involved group (n = 339)	*T-value*
Total score	127.78±17.94	95.87±21.99	-22.575***	121.14±21.08	100.44±26.05	-12.247***	120.59±20.70	99.24±26.67	-12.283***
F1	3.23±0.87	2.36±0.81	-14.614***	3.04±0.90	2.50±0.92	-8.490***	3.05±0.88	2.42±0.92	-9.782***
F2	3.72±0.70	2.82±0.87	-16.178***	3.52±0.81	2.97±0.93	-8.806***	3.46±0.80	3.00±0.98	-7.120***
F3	3.27±0.97	2.32±0.93	-14.032***	3.02±1.03	2.51±1.03	-6.954***	3.07±1.01	2.40±1.01	-9.167***
F4	3.63±0.76	2.61±0.92	-17.220***	3.45±0.89	2.72±0.95	-11.274***	3.41±0.86	2.70±1.00	-10.587***
F5	4.14±0.57	3.41±0.86	-14.096***	4.02±0.66	3.47±0.89	-9.796***	3.99±0.67	3.47±0.90	-8.985***
F6	3.62±0.86	2.79±0.98	-12.735***	4.02±0.66	3.47±0.89	-9.796***	3.46±0.92	2.85±1.03	-8.744***
F7	2.99±1.09	2.04±0.92	-13.279***	2.74±1.12	2.24±1.04	-6.548***	2.76±1.11	2.16±1.02	-8.004***
F8	3.63±082	2.68±0.94	-15.256***	3.48±0.87	2.77±1.02	-10.525***	3.45±0.87	2.74±1.02	-10.353***
F9	3.74±0.74	2.94±0.95	-13.172***	3.56±0.87	3.07±0.96	-7.517***	3.54±0.85	3.06±0.99	-7.218***

Similarly, fans were divided into high and low groups according to their total scores on the excessive celebrity worship behavior scale. There were significant differences between the two groups for both the average daily duration of time (T) and the average daily frequency of using mobile phones for celebrity worship (F), as shown in Table 9.

**Table 9:** Discriminative validity of excessive celebrity worship behavior scale on the duration of time and frequency of online celebrity worship.

Project	High score group (n = 407)	Low score group (n = 397)	*T-value*	*df*	*P value*
Ta	4.44±1.510	3.10±1.548	12.437	802	<0.001
Fb	2.08±0.799	1.52±0.740	12.283	613.686	<0.001

Note: a. The daily duration is scored by 7 points, which are 1 = 10 minutes-0.5 hours, 2 = 0.5–1 hours, 3 = 1–1.5 hours, 4 = 1.5–2 hours, 5 = 2–3 hours, 6 = 3–4 hours and 7 = more than 4 hours. b. Daily frequency was scored on 4 points, which are 1 = 0–10 times, 2 = 11–20 times, 3 = 21–50 times, and 4 = 50 times.

### 3.7 Scoring by demographics

The results showed that the total score ranged from 36 to 178, the average score for each factor ranged from 1 to 4.94, and the mean value and standard deviation were 3.10 ± 0.71. The results of the paired t-test showed that among the nine factors, the score of mood alteration was the highest, followed by escape from real life and replacement of real to virtual social relationships. The score for excessive buying was the lowest, followed by impaired social functioning and sleep and eating problems. Comparing the gender differences, it was found that there was no significant difference in the total score; however, in the factors of impaired social functioning, replacement of real to virtual social relationships, and excessive buying, male fans scored significantly higher than female fans, while female fans scored higher than male fans in terms of mood alteration, showing marginal significance. In terms of the age difference, the total score and several factors showed that the level of excessive behavior of fans over 23 years old was higher than that of fans under 23 years old. The results are summarized in Table 10.

**Table 10:** Scores and differences in gender and age.

		Mean ± SD	Gender Independent sample t-test			Age ANOVA	
			Male	Female	*T-value*	*F*	Pairwise comparison
Total score		111.59±25.66	114.04±25.25	110.86±25.75	1.475	4.394**	2,3,4>1
F1	Impaired social functioning	2.79±0.95	2.95±0.94	2.74±0.94	2.637**	1.456	-
F2	Replacement of real to virtual social relationships	3.27±0.91	3.37±0.81	3.23±0.93	1.979*	10.490***	4>2,3>1
F3	Sleep and eating problems	2.79±1.06	2.90±1.05	2.75±1.06	1.601	3.749*	3,4>1
F4	Withdrawal	3.11±0.99	3.16±0.94	3.10±1.00	0.773	5.107**	2,3>1
F5	Mood alteration	3.77±0.82	3.67±0.82	3.79±0.82	-1.745†	4.378**	2,3,4>1
F6	Salience	3.20±1.01	3.13±0.97	3.22±1.03	-1.158	1.282	-
F7	Excessive buying	2.51±1.11	2.78±1.08	2.43±1.11	3.737***	2.045	-
F8	Increased craving	3.15±1.00	3.23±1.02	3.13±1.00	1.236	3.137*	3,4>1
F9	Escape from real-life	3.33±0.94	3.25±0.90	3.36±0.95	-1.346	0.484	-

Note: Age groups are 1 = 23 years old and below; 2 = 24 ~ 26 years old; 3 = 27 ~ 30 years old; and 4 = above 30 years old.

## 4. Discussion

Based on Internet addiction, problematic Internet use, smartphone addiction, and other scales [47, 48], this study developed a measurement scale specifically for excessive celebrity worship behavior of young fans. This scale reflects the multidimensional characteristics of fans’ addictive behavior. The results showed that the scale had nine factors: impaired social functioning, replacement of real to virtual social relationships, sleep and eating problems, withdrawal, mood alteration, salience, excessive buying, increased craving, and escape from real life, and it had good reliability and validity.

Existing studies have shown that absorption-addiction model has three components: entertainment-social, intense–personal, and borderline–pathological. High levels of celebrity worship can be associated with multiple negative outcomes such as depression, anxiety, addictive tendencies, stalking, compulsive buying, social dysfunction, and violation of law etc. [26]. The rapid development of new media technology and pop culture has greatly promoted the formation of fandom culture in East Asia, in which fans are deeply involved, and excessive behavior intertwines with problematic smartphone/social media use. Among the nine factors of our scale, impaired social functioning, sleep and eating problems, and excessive buying were highly correlated, which were higher than other factors; therefore, many items were removed because of the high cross-loading of these three factors. These factors may match the negative consequences, social and health problems, or time management problems in previous scales [49] and can be deemed as the negative spillover effect of celebrity worship on daily fundamental activities. According to the exploratory factor analysis results in this study, although they were intercorrelated, there were significant differences among the three. Consumption, sleep/eating, and interpersonal relationship belong to different types of daily activities in terms of theoretical concept connotation. Therefore, this study extracted impaired social functioning, sleep and eating problems, and excessive buying into three factors that conformed to the data structure and were consistent with the theoretical structure. The four factors: salience, increased craving, replacement of real to virtual social relationships, and withdrawal on this scale kept robust on structure [50]. In addition to the data structure, the theoretical structure is an important criterion for this scale. In the factor of excessive buying, the item "I have concealed the real-time I spent following idols" was categorized together with the items of excessive buying in preliminary EFA. If the factor were named "loss of control,” it would cover up the essence that the factor was mainly composed of excessive consumption, so the single item was removed to make sure the factor reflected the impulsivity and loss of control on consumption behavior caused by celebrity worship [51]. Therefore, both the data and theoretical structures are important criteria for developing this scale.

Differences in gender and age were also examined in this study. Our preliminary data showed that the proportion of female fans was higher than that of male fans, which is consistent with previous studies. It was reported that, in China, the number/proportion of female celebrity/pop-idol fans largely outruns male ones [8, 51]. This is also consistent with researches in some other regions [19, 20]. There have been heterogeneous results in the existing studies in terms of behavioral patterns. Some studies found that females scored higher than males on the CAS overall [52], the entertainment-social subscale [53, 54], the intense-personal subscale [55, 56], or borderline pathology with student sample; some reported that males showed higher scores on total CAS [19], entertainment-social [19], intense-personal [57], or borderline-pathological CW [58, 59], for example, celebrity stalking [18]. Studies on Chinese fans have revealed that female fans are more devoted to and obsessed with celebrity worship [60] and prone to show romantic attachment and high consumption [61]. Some studies found no significant evidence of gender differences [6, 62–65]. In our study, there was no significant gender difference in the total score on the scale. However, male fans may have higher social functioning, replacement of real to virtual social relationships, and excessive buying behavior than female fans. This is consistent with previous studies reporting higher scores for borderline pathological CW among male fans. Female fans had higher mood alteration than male fans. In summary, our results indicated that the level of pathological or problematic behavior seems similar in male and female fans, but may differ in certain aspects. Therefore, when Internet addiction and celebrity worship are intertwined, gender differences become more complex.

Previous studies on age-related differences in Internet addiction have yielded inconsistent conclusions. Some believe that younger individuals are more likely to become addicted, such as 17–18 years old adolescents have better self-control than 12–16 years old adolescents [66], while others believe that the degree of addiction increases with age because the prevalence of screen devices and flexible time is higher and the level of parental control is lower. Some studies have reported that age differences are not significant [67]. In the studies of celebrity worship, some believed that the worship behavior reaches its peak at the age of 14–16, showing a significant downward trend from junior high school students to college students [60] or at the age of early adolescence (age 10–11) [20], or in middle adolescence [27], or in older adolescents with borderline-pathological CW [59]. In adults, age was positively correlated with intense personal CW and negatively correlated with entertainment-social and borderline-pathological CW [68]. Other studies found no significant age difference, probably because of the high prevalence and development of social media and the pop industry [6, 9, 69, 70]. The data of this study indicated that fans over the age of 23 showed a higher level of excessive behavior than younger fans in terms of the total score and several factors. The underlying mechanism might be that, on the one hand, older adults have already graduated from school and are financially independent, and therefore showed stronger resource mobilization capability and usually occupied dominant position in the fandom which was associated with deeper involvement. They have more autonomy in managing their own lives and, therefore, may be more likely to have sleep and eating problems or withdraw when they lack self-control. On the other hand, older adults are facing greater social competition and mental stress, so using mobile phones and new media platforms for celebrity worship can produce stronger virtual social relationships and mood alteration. These results also showed that age can be a complicated factor when internet addiction and celebrity worship are intertwined. However, there were very few middle school students under the age of 18 years in the sample of this study; therefore, it is difficult to compare the problematic starchasing behavior of this group.

In sum, this study mainly has two theoretical contributions. For one thing, in terms of celebrity worship, this study systematically introduced the theoretical lens of behavioral addiction to examine super fans’ behavior. The scales of problematic Internet use have been rapidly developing and thus this research can consolidate the theoretical foundation in this regard. By reviewing previous studies, it was clarified that the most widely adopted absorption-addiction model organized the components on three successive levels and only a few items of it measured problematic behavior of fans. IEI and some other scales consider idolatry to be a normal part of everyone and measure parasocial behavior from a beneficial or neutral perspective, for instance, attachment, consumption, identification, idealization, and romanticization [9]. The Obsessive Relational Intrusion and Celebrity Stalking Scale mainly describes the specific type of behavior of sasaeng fans [18]. In this way, the current study integrated a new theoretical lens different from the above to systematically examine excessive celebrity worship behavior. For another, from the perspective of problematic Internet use and behavioral addiction, this scale is developed for a specific context—celebrity worship and it can identify nine factors within this context. The nine factors overlap with the concepts from multiple existing scales of problematic Internet use, quantitatively confirming that excessive celebrity worship behavior shows an addictive pattern as predicted in previous studies. Despite this, there are still a few noteworthy aspects. For example, “sleep and eating problems” in the scale of this research becomes a separate factor from “Interference with daily life” [71], indicating the significance of such problems in fans; “neglecting duties” “social relationship disruption” and “social interactive problems” all appear in the same factor [72–74] which is named “impaired social functioning” in this scale, indicating a core element of “neglecting” in fans’ behavioral pattern; “increased craving” in the scale includes items that reflect the “time management problem” and “loss of control” [75]. The framework of this research unveiled that the maladaptive behavior of online celebrity worship may have a pattern different from other problematic behaviors, providing a research tool for future empirical studies on cyber subculture and youth social media use.

However, some limitations need to be addressed in future research. First, the scale needs more validation, e.g. the effectiveness of face validity could be raised with quantitative test. It could also be improved with larger samples, other cultures and languages, and a larger number of adolescent participants. Second, according to previous studies, demographic variables may have complicated interactions. For example, gender differences may vary along with age. Therefore, further studies are needed to explore these interactions. Third, this new scale can be used in future studies to examine whether excessive celebrity worship is associated with group-level fandom performance, such as leadership and perceived organizational politics.

## Supporting information

S1 Dataset (XLSX)

S2 Dataset (XLSX)
